# 1-(4-Chloro-2-fluoro­phen­yl)-4-difluoro­methyl-3-methyl-1*H*-1,2,4-triazol-5(4*H*)-one

**DOI:** 10.1107/S1600536812009452

**Published:** 2012-03-14

**Authors:** Dong-mei Ren, Yong-yi Wang

**Affiliations:** a Security and Environment Engineering College, Capital University of Economics and Business, Beijing 10070, People’s Republic of China

## Abstract

In the crystal structure of the title compound, C_10_H_7_ClF_3_N_3_O, pairs of mol­ecules are connected into dimers *via* pairs of C—H⋯O hydrogen bonds. The dihedral angle between the benzene ring and attached triazolone ring is 53.2 (1)°.

## Related literature

For background to this class of compound, see: Ager & Polz (1996 ▶); Li & Han (2010 ▶). For the synthesis of the title compound, see: Jaidev & Plainsboro (1998 ▶). For bond-length data, see: Allen *et al.* (1987 ▶).
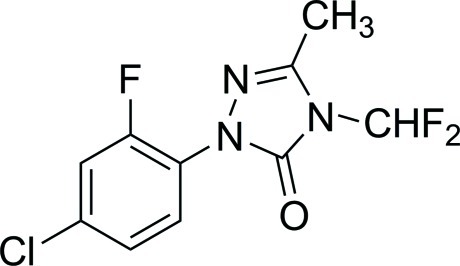


## Experimental

### 

#### Crystal data

C_10_H_7_ClF_3_N_3_O
*M* *_r_* = 277.64
Monoclinic, 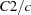

*a* = 15.286 (3) Å
*b* = 13.610 (3) Å
*c* = 11.231 (2) Å
β = 100.91 (3)°
*V* = 2294.3 (8) Å^3^
*Z* = 8
Mo *K*α radiation
μ = 0.36 mm^−1^
*T* = 293 K
0.30 × 0.20 × 0.10 mm

#### Data collection

Enraf–Nonius CAD-4 diffractometer
Absorption correction: ψ scan (North *et al.*, 1968 ▶) *T*_min_ = 0.899, *T*_max_ = 0.965
4290 measured reflections
2115 independent reflections
1273 reflections with *I* > 2σ(*I*)
*R*_int_ = 0.036
3 standard reflections every 200 reflections intensity decay: 1%

#### Refinement

*R*[*F* ^2^ > 2σ(*F* ^2^)] = 0.050
*wR*(*F* ^2^) = 0.143
*S* = 1.00
2115 reflections
164 parameters
H-atom parameters constrained
Δρ_max_ = 0.23 e Å^−3^
Δρ_min_ = −0.34 e Å^−3^

### 

Data collection: *CAD-4 Software* (Enraf–Nonius, 1985 ▶); cell refinement: *CAD-4 Software*; data reduction: *XCAD4* (Harms & Wocadlo, 1995 ▶); program(s) used to solve structure: *SHELXS97* (Sheldrick, 2008 ▶); program(s) used to refine structure: *SHELXS97* (Sheldrick, 2008 ▶); molecular graphics: *SHELXTL* (Sheldrick, 2008 ▶); software used to prepare material for publication: *SHELXTL*.

## Supplementary Material

Crystal structure: contains datablock(s) I, global. DOI: 10.1107/S1600536812009452/bq2341sup1.cif

Structure factors: contains datablock(s) I. DOI: 10.1107/S1600536812009452/bq2341Isup2.hkl

Supplementary material file. DOI: 10.1107/S1600536812009452/bq2341Isup3.cml

Additional supplementary materials:  crystallographic information; 3D view; checkCIF report

## Figures and Tables

**Table 1:** Hydrogen-bond geometry (Å, °)

*D*—H⋯*A*	*D*—H	H⋯*A*	*D*⋯*A*	*D*—H⋯*A*
C10—H10*A*⋯O^i^	0.98	2.41	3.259 (4)	144

Symmetry code: (i) 
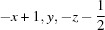
.

**Fig. 1.**
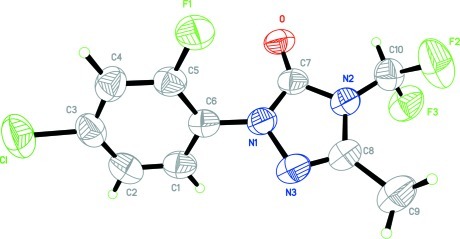
The molecular structure of (I), with the atom-numbering scheme. Displacement ellipsoids are drawn at the 50% probability level.

**Fig. 2.**
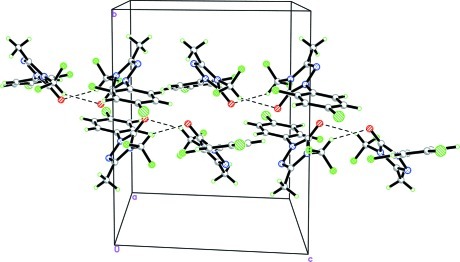
A packing diagram of (I) showing the dimers formed by C—H···O H-bonds.

## supplementary crystallographic information

### Comment

The title compound is an important intermediate used to synthesize the Carfentrazone-ethyl, which can be utilized to synthesize herbicides (Jaidev & Plainsboro, 1998), which are of wide interest for applications in control of broadleaf weeds and sedges (Ager & Polz, 1996). They are widely used in protection of wheat, barley, oats, rice, corn, *etc* (Li & Han, 2010). We report here the crystal structure of the title compound, (I), which is of interest to us in the field.

The molecular structure of (I) is shown in Figure 1. In the structure, the molecules were connected together *via* C—H···O intermolecular hydrogen bonds (Table 1 and Figure 2.) to form dimers. The dihedral angle of the rings A(C1—C6), B(N1/N3/C8/N2/C7) is: A/B = 53.2 (1)°.

### Experimental

The title compound, (I) was prepared by a method reported in literature (Jaidev & Plainsboro, 1998). The crystals were obtained by dissolving (I) (0.2 g) in acetone (50 ml) and evaporating the solvent slowly at room temperature for about 10 d.

### Refinement

All H atoms were positioned geometrically and constrained to ride on their parent atoms, with C—H = 0.93 Å for aromatic H and 0.96 Å for alkyl H. The *U*_iso_(H) = *xU*_eq_(C), where *x* = 1.2 for aromatic H, and *x* = 1.5 for alkyl H.

### Crystal data

**Table:**

C_10_H_7_ClF_3_N_3_O	*F*(000) = 1120
*M**_r_* = 277.64	*D*_x_ = 1.608 Mg m^−^^3^
Monoclinic, *C*2/*c*	Mo *K*α radiation, λ = 0.71073 Å
Hall symbol: -C 2yc	Cell parameters from 25 reflections
*a* = 15.286 (3) Å	θ = 10–13°
*b* = 13.610 (3) Å	µ = 0.36 mm^−^^1^
*c* = 11.231 (2) Å	*T* = 293 K
β = 100.91 (3)°	Block, colorless
*V* = 2294.3 (8) Å^3^	0.30 × 0.20 × 0.10 mm
*Z* = 8	

### Data collection

**Table:**

Enraf–Nonius CAD-4 diffractometer	1273 reflections with *I* > 2σ(*I*)
Radiation source: fine-focus sealed tube	*R*_int_ = 0.036
Graphite monochromator	θ_max_ = 25.4°, θ_min_ = 2.0°
ω/2θ scans	*h* = 0→18
Absorption correction: ψ scan (North *et al.*, 1968)	*k* = −16→16
*T*_min_ = 0.899, *T*_max_ = 0.965	*l* = −13→13
4290 measured reflections	3 standard reflections every 200 reflections
2115 independent reflections	intensity decay: 1%

### Refinement

**Table:**

Refinement on *F*^2^	Primary atom site location: structure-invariant direct methods
Least-squares matrix: full	Secondary atom site location: difference Fourier map
*R*[*F*^2^ > 2σ(*F*^2^)] = 0.050	Hydrogen site location: inferred from neighbouring sites
*wR*(*F*^2^) = 0.143	H-atom parameters constrained
*S* = 1.00	*w* = 1/[σ^2^(*F*_o_^2^) + (0.073*P*)^2^] where *P* = (*F*_o_^2^ + 2*F*_c_^2^)/3
2115 reflections	(Δ/σ)_max_ < 0.001
164 parameters	Δρ_max_ = 0.23 e Å^−^^3^
0 restraints	Δρ_min_ = −0.34 e Å^−^^3^

### Special details

**Table:**

Geometry. All e.s.d.'s (except the e.s.d. in the dihedral angle between two l.s. planes) are estimated using the full covariance matrix. The cell e.s.d.'s are taken into account individually in the estimation of e.s.d.'s in distances, angles and torsion angles; correlations between e.s.d.'s in cell parameters are only used when they are defined by crystal symmetry. An approximate (isotropic) treatment of cell e.s.d.'s is used for estimating e.s.d.'s involving l.s. planes.
Refinement. Refinement of *F*^2^ against ALL reflections. The weighted *R*-factor *wR* and goodness of fit *S* are based on *F*^2^, conventional *R*-factors *R* are based on *F*, with *F* set to zero for negative *F*^2^. The threshold expression of *F*^2^ > σ(*F*^2^) is used only for calculating *R*-factors(gt) *etc*. and is not relevant to the choice of reflections for refinement. *R*-factors based on *F*^2^ are statistically about twice as large as those based on *F*, and *R*- factors based on ALL data will be even larger.

### Fractional atomic coordinates and isotropic or equivalent isotropic displacement parameters (Å^2^)

**Table:**

	*x*	*y*	*z*	*U*_iso_*/*U*_eq_	
Cl	0.07385 (7)	0.56530 (8)	0.15418 (11)	0.1001 (5)	
O	0.44175 (13)	0.54039 (17)	−0.12275 (18)	0.0621 (6)	
F1	0.26705 (12)	0.64405 (16)	−0.14770 (16)	0.0796 (6)	
N1	0.42189 (15)	0.65403 (18)	0.0253 (2)	0.0529 (6)	
C1	0.3335 (2)	0.6085 (2)	0.1748 (3)	0.0637 (9)	
H1A	0.3846	0.6094	0.2348	0.076*	
N2	0.54325 (16)	0.66315 (18)	−0.0433 (2)	0.0541 (6)	
F2	0.62510 (18)	0.72262 (17)	−0.1740 (2)	0.1082 (8)	
C2	0.2523 (3)	0.5883 (3)	0.2052 (3)	0.0716 (10)	
H2A	0.2483	0.5761	0.2855	0.086*	
F3	0.68716 (13)	0.62152 (16)	−0.0370 (2)	0.0902 (7)	
N3	0.47231 (18)	0.72846 (19)	0.0904 (2)	0.0598 (7)	
C3	0.1768 (2)	0.5862 (2)	0.1157 (3)	0.0650 (9)	
C4	0.1812 (2)	0.6026 (2)	−0.0036 (3)	0.0643 (9)	
H4A	0.1304	0.5994	−0.0639	0.077*	
C5	0.2627 (2)	0.6237 (2)	−0.0314 (3)	0.0547 (8)	
C6	0.3396 (2)	0.6273 (2)	0.0557 (3)	0.0519 (7)	
C7	0.4651 (2)	0.6095 (2)	−0.0559 (3)	0.0519 (7)	
C8	0.5439 (2)	0.7331 (2)	0.0472 (3)	0.0576 (8)	
C9	0.6171 (3)	0.8030 (3)	0.0887 (4)	0.0860 (11)	
H9A	0.6034	0.8418	0.1541	0.129*	
H9B	0.6242	0.8454	0.0228	0.129*	
H9C	0.6714	0.7674	0.1160	0.129*	
C10	0.6099 (2)	0.6433 (3)	−0.1112 (3)	0.0653 (9)	
H10A	0.5914	0.5883	−0.1667	0.078*	

### Atomic displacement parameters (Å^2^)

**Table:**

	*U*^11^	*U*^22^	*U*^33^	*U*^12^	*U*^13^	*U*^23^
Cl	0.0851 (7)	0.0982 (8)	0.1308 (10)	−0.0103 (6)	0.0553 (7)	−0.0134 (7)
O	0.0627 (13)	0.0687 (14)	0.0523 (12)	−0.0061 (11)	0.0041 (10)	−0.0161 (11)
F1	0.0680 (12)	0.1151 (16)	0.0514 (11)	0.0087 (11)	0.0005 (9)	0.0147 (10)
N1	0.0493 (14)	0.0558 (15)	0.0515 (14)	−0.0005 (12)	0.0043 (11)	−0.0083 (12)
C1	0.072 (2)	0.067 (2)	0.0484 (19)	0.0064 (17)	0.0025 (16)	−0.0009 (15)
N2	0.0524 (15)	0.0542 (15)	0.0540 (15)	−0.0009 (12)	0.0059 (12)	−0.0041 (12)
F2	0.133 (2)	0.0924 (17)	0.1152 (18)	0.0002 (15)	0.0645 (16)	0.0242 (14)
C2	0.090 (3)	0.068 (2)	0.061 (2)	0.006 (2)	0.026 (2)	0.0009 (17)
F3	0.0532 (12)	0.1008 (17)	0.1121 (17)	0.0055 (11)	0.0040 (11)	−0.0142 (13)
N3	0.0555 (16)	0.0563 (17)	0.0631 (17)	0.0009 (13)	0.0000 (13)	−0.0123 (12)
C3	0.068 (2)	0.0554 (19)	0.078 (2)	0.0011 (16)	0.0301 (19)	−0.0070 (17)
C4	0.051 (2)	0.065 (2)	0.074 (2)	0.0058 (16)	0.0060 (17)	−0.0029 (17)
C5	0.0574 (19)	0.0596 (19)	0.0447 (18)	0.0100 (15)	0.0033 (14)	0.0038 (14)
C6	0.0565 (19)	0.0483 (17)	0.0492 (18)	0.0060 (14)	0.0054 (14)	−0.0016 (13)
C7	0.0523 (18)	0.0570 (19)	0.0428 (17)	0.0052 (15)	0.0000 (14)	0.0011 (15)
C8	0.0539 (19)	0.0500 (18)	0.065 (2)	0.0000 (15)	0.0003 (16)	−0.0046 (15)
C9	0.081 (3)	0.068 (2)	0.105 (3)	−0.012 (2)	0.007 (2)	−0.020 (2)
C10	0.062 (2)	0.067 (2)	0.069 (2)	−0.0001 (17)	0.0165 (18)	0.0006 (17)

### Geometric parameters (Å, º)

**Table:**

Cl—C3	1.733 (3)	C2—C3	1.380 (5)
O—C7	1.214 (3)	C2—H2A	0.9300
F1—C5	1.349 (3)	F3—C10	1.344 (4)
N1—C7	1.365 (4)	N3—C8	1.280 (4)
N1—N3	1.393 (3)	C3—C4	1.373 (4)
N1—C6	1.412 (4)	C4—C5	1.371 (4)
C1—C2	1.375 (5)	C4—H4A	0.9300
C1—C6	1.383 (4)	C5—C6	1.380 (4)
C1—H1A	0.9300	C8—C9	1.476 (5)
N2—C7	1.385 (4)	C9—H9A	0.9600
N2—C8	1.391 (4)	C9—H9B	0.9600
N2—C10	1.409 (4)	C9—H9C	0.9600
F2—C10	1.334 (4)	C10—H10A	0.9800
			
C7—N1—N3	112.6 (2)	C5—C6—C1	118.3 (3)
C7—N1—C6	127.8 (3)	C5—C6—N1	121.1 (3)
N3—N1—C6	119.4 (2)	C1—C6—N1	120.5 (3)
C2—C1—C6	120.4 (3)	O—C7—N1	129.4 (3)
C2—C1—H1A	119.8	O—C7—N2	128.2 (3)
C6—C1—H1A	119.8	N1—C7—N2	102.4 (3)
C7—N2—C8	108.8 (3)	N3—C8—N2	110.8 (3)
C7—N2—C10	122.8 (3)	N3—C8—C9	124.3 (3)
C8—N2—C10	128.4 (3)	N2—C8—C9	124.9 (3)
C1—C2—C3	119.5 (3)	C8—C9—H9A	109.5
C1—C2—H2A	120.2	C8—C9—H9B	109.5
C3—C2—H2A	120.2	H9A—C9—H9B	109.5
C8—N3—N1	105.3 (2)	C8—C9—H9C	109.5
C4—C3—C2	121.3 (3)	H9A—C9—H9C	109.5
C4—C3—Cl	119.0 (3)	H9B—C9—H9C	109.5
C2—C3—Cl	119.7 (3)	F2—C10—F3	106.6 (3)
C5—C4—C3	118.1 (3)	F2—C10—N2	110.3 (3)
C5—C4—H4A	120.9	F3—C10—N2	110.3 (3)
C3—C4—H4A	120.9	F2—C10—H10A	109.9
F1—C5—C4	118.5 (3)	F3—C10—H10A	109.9
F1—C5—C6	119.2 (3)	N2—C10—H10A	109.9
C4—C5—C6	122.3 (3)		
			
C6—C1—C2—C3	0.5 (5)	N3—N1—C7—O	176.9 (3)
C7—N1—N3—C8	2.6 (3)	C6—N1—C7—O	3.1 (5)
C6—N1—N3—C8	177.0 (2)	N3—N1—C7—N2	−3.1 (3)
C1—C2—C3—C4	0.9 (5)	C6—N1—C7—N2	−177.0 (2)
C1—C2—C3—Cl	−177.6 (3)	C8—N2—C7—O	−177.6 (3)
C2—C3—C4—C5	−1.7 (5)	C10—N2—C7—O	0.2 (5)
Cl—C3—C4—C5	176.9 (2)	C8—N2—C7—N1	2.5 (3)
C3—C4—C5—F1	−177.1 (3)	C10—N2—C7—N1	−179.8 (3)
C3—C4—C5—C6	1.0 (5)	N1—N3—C8—N2	−0.8 (3)
F1—C5—C6—C1	178.5 (3)	N1—N3—C8—C9	179.4 (3)
C4—C5—C6—C1	0.3 (5)	C7—N2—C8—N3	−1.1 (3)
F1—C5—C6—N1	1.4 (4)	C10—N2—C8—N3	−178.7 (3)
C4—C5—C6—N1	−176.8 (3)	C7—N2—C8—C9	178.7 (3)
C2—C1—C6—C5	−1.1 (5)	C10—N2—C8—C9	1.1 (5)
C2—C1—C6—N1	176.1 (3)	C7—N2—C10—F2	122.1 (3)
C7—N1—C6—C5	−58.7 (4)	C8—N2—C10—F2	−60.6 (4)
N3—N1—C6—C5	127.8 (3)	C7—N2—C10—F3	−120.5 (3)
C7—N1—C6—C1	124.2 (3)	C8—N2—C10—F3	56.8 (4)
N3—N1—C6—C1	−49.3 (4)		

### Hydrogen-bond geometry (Å, º)

**Table:**

*D*—H···*A*	*D*—H	H···*A*	*D*···*A*	*D*—H···*A*
C10—H10*A*···O^i^	0.98	2.41	3.259 (4)	144

Symmetry code: (i) −*x*+1, *y*, −*z*−1/2.
